# Engineering Structurally Interacting RNA (sxRNA)

**DOI:** 10.1038/srep45393

**Published:** 2017-03-28

**Authors:** Francis Doyle, Sameer Lapsia, Salvatore Spadaro, Zachary E. Wurz, Sumita Bhaduri-McIntosh, Scott A. Tenenbaum

**Affiliations:** 1 Nanobioscience Constellation, College of Nanoscale Science and Engineering, SUNY Polytechnic Institute, Albany, NY New York 12203, USA; 2 Department of Pediatrics, Stony Brook University School of Medicine, Stony Brook, NY 11794, USA; 3 HocusLocus, LLC, 253 Fuller Road, Nanofab North, Albany NY 12203, USA; 4 Pediatric Infectious Diseases, Departments of Pediatrics and Molecular Genetics and Microbiology, Stony Brook University School of Medicine, Stony Brook, NY 11794, USA

## Abstract

RNA-based three-way junctions (3WJs) are naturally occurring structures found in many functional RNA molecules including rRNA, tRNA, snRNA and ribozymes. 3WJs are typically characterized as resulting from an RNA molecule folding back on itself in *cis* but could also form in *trans* when one RNA, for instance a microRNA binds to a second structured RNA, such as a mRNA. *Trans-*3WJs can influence the final shape of one or both of the RNA molecules and can thus provide a means for modulating the availability of regulatory motifs including potential protein or microRNA binding sites. Regulatory 3WJs generated in *trans* represent a newly identified regulatory category that we call structurally interacting RNA or sxRNA for convenience. Here we show that they can be rationally designed using familiar *cis-*3WJ examples as a guide. We demonstrate that an sxRNA “bait” sequence can be designed to interact with a specific microRNA “trigger” sequence, creating a regulatable RNA-binding protein motif that retains its functional activity. Further, we show that when placed downstream of a coding sequence, sxRNA can be used to switch “ON” translation of that sequence in the presence of the trigger microRNA and the amount of translation corresponded with the amount of microRNA present.

The established RNA three-way junction (3WJ) structural element results from a single RNA molecule folding back on itself in a *cis-*fashion, to produce three separate helices that meet around a central unpaired hub-region. The 3WJ is an important structural component of a wide variety of non-coding RNAs including most notably, ribosomal RNAs, which contain numerous examples^1^. The occurrence of 3WJs in complex structural RNAs is considered necessary to achieve the particular relative orientation of specific subunits, allowing and possibly maximizing essential function and interactions with RNA-binding proteins (RBPs)^2^. Recent research also suggests that 3WJs may lend a particularly high stability to these essential conformations^3^. Multiple studies have recently demonstrated that there is an abundance of previously unrecognized RNA-RNA interactions that occur in *trans* and that many have the potential for significant regulatory importance^4^,^5^,^6^. We previously showed that the classic 3WJ structure could be formed from the unconventional binding, in *trans,* of a microRNA to a mRNA producing a stem-loop structure that could be targeted by an RBP^7^. We suggested that *trans*-3WJs could serve as the foundation for designing regulatable RNA “switches” that would form only in the presence of a particular microRNA and would recruit a regulatory RBP that controls protein translation^7^,^8^.

Many RBPs associate with their RNA targets by binding to motifs with structures that contain one or more stem-loops^9^,^10^,^11^. To further develop the sxRNA technology, we focused on one of the best characterized examples of this, the histone stem-loop binding protein (SLBP), which binds to the histone stem-loop (HSL) motif located at the 3′-end of most metazoan histone mRNAs and is essential for the proper post-transcriptional regulation and translation of this critical family of proteins^12^,^13^,^14^. Histone proteins play an essential role in regulating transcriptional activity and gene expression by packaging DNA into chromatin^15^. There are five major families of histones and their mRNAs are unique in that they are the only naturally occurring eukaryotic mRNAs that rarely have poly-A tails^16^. Instead, the regulation of metazoan histones requires a specialized post-transcriptional mechanism using the conserved HSL motif comprised of a 16-base stem-loop structure plus 3–4 bases on both its immediate 5′ and 3′ flanks. The HSL is found at the extreme 3′-end of histone mRNA and when bound by SLBP, stabilizes the message and greatly increases its translation by as much as an order of magnitude or more^12^,^13^,^14^,^16^. Significantly, studies have demonstrated that when mRNA containing a terminal HSL is ectopically introduced into cells, it will be actively translated upon endogenous SLBP binding, and can be used to efficiently express a reporter protein^14^,^17^. Additionally, it has been shown that point mutations to the HSL sequence, affecting SLBP affinity, lead to varying levels of expression of these constructs^17^.

Using an informatic-based approach, we previously predicted potential naturally occurring *trans-*3WJ structures that may result from the atypical binding of microRNAs to the flanking region at the base of the HSL-motif and termed these complexes, *structurally interacting RNAs* or “sxRNA” for short^7^. Using the recognized rules for *cis-*3WJ structures^1^, we demonstrate the ability to rationally design a two-piece, *trans* HSL-sxRNA into a reporter message, with a regulatable, HSL-sequence (the bait) that requires a targeted cellular microRNA to act as a “trigger” to form a functional *trans*-HSL motif, which can now associate with endogenous SLBP. We show that an HSL-sxRNA can be used to regulate the translation of an upstream coding sequence and that expression correlates with the amount of triggering microRNA present in the cell.

## Results

### Designing *trans-*3WJs

The ability to re-engineer *cis*-3WJs to form in *trans* has been demonstrated^3^,^18^,^19^,^20^,^21^,^22^. For example, the phi29 bacteriophage contains an RNA 3WJ, which functions as part of its DNA packaging motor. This structure has been shown to form from three synthetically produced independent RNA strands. Using the recognized description for 3WJs^1^, we developed an approach for engineering and labeling *trans-3*WJ*s* interactions. Existing definitions of 3WJs categorize structures into three general families based on characteristics relative to the two roughly coaxially stacked helices central to the 3WJ. While it is difficult to accurately predict which of the *trans* formed 3WJ helices will orient in a defined manner, generally we have sought to produce “Family A” type 3WJ^1^, where the two coaxial helices are those formed by the direct base-pairing of the *trans*-RNA. The intent behind this approach was to produce a structure where no two helices would be forced into such close proximity that one might block the availability for protein binding.

**Figure 1:**
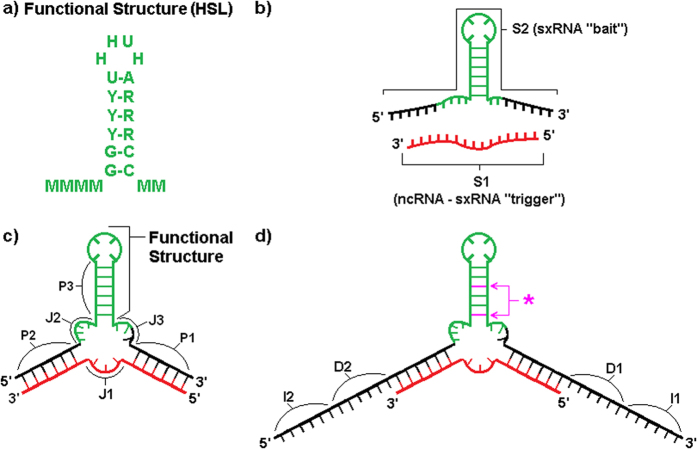
sxRNA Design Schematic. (**a**) Consensus sequence for the human histone stem-loop (HSL) motif^10^,^42^. (**b**) A generic 3WJ sxRNA is depicted detailing various constituent parts. S1 (“strand 1”, in red) denotes the targeted, *trans* activating “trigger” ncRNA. S2 denotes the engineered sxRNA “bait” sequence. (**c**) The same sequences from (**b**) are depicted detailing various constituent parts. P1-P3 represents the “paired regions” constituting the three helices of the 3WJ. P3 is the reinforced sxRNA stem of the switched functional structure (shown in green). J1-J3 are the unpaired “junction regions” of the 3WJ. (**d**) Purple asterisk and arrows point to optional non-canonical base pairs, which may be introduced at various positions to weaken structure in absence of S1. D1-D2 denotes optional “destabilizing” sequences that may be introduced to promote alternative conformations in absence of S1. I1-I2 denotes optional “isolation” sequences that may be introduced to prevent switch regions from interacting with the surrounding RNA sequence.

We designed our sxRNA constructs to incorporate a wild type or consensus HSL-sequence (Fig. 1a) as one of the three helices. In all cases, this functional helix is fully contained within a single artificially designed sequence we termed the “bait” (Fig. 1 and Supplementary Figs S1–S6, depicted in green). The remaining two helices of the 3WJ were engineered to form based on conventional Watson-Crick base-pairing of the bait sequence to a target microRNA of interest. The target microRNA, which we term the “trigger”, represents the second RNA strand in the *trans* interaction (Fig. 1 and Supplementary Figs S1–S6, depicted in red). Once initial designs were selected, we used RNAcofold^23^ to confirm that the informatically predicted interaction recapitulated the desired helices. Table 1 provides the annotation and sequences used for the sxRNA bait and triggers used in this study. Supplementary Table S1 provides a more comprehensive description of these sequences with additional information.

### *In-vitro* binding of *trans*-3WJ

**Figure 2:**
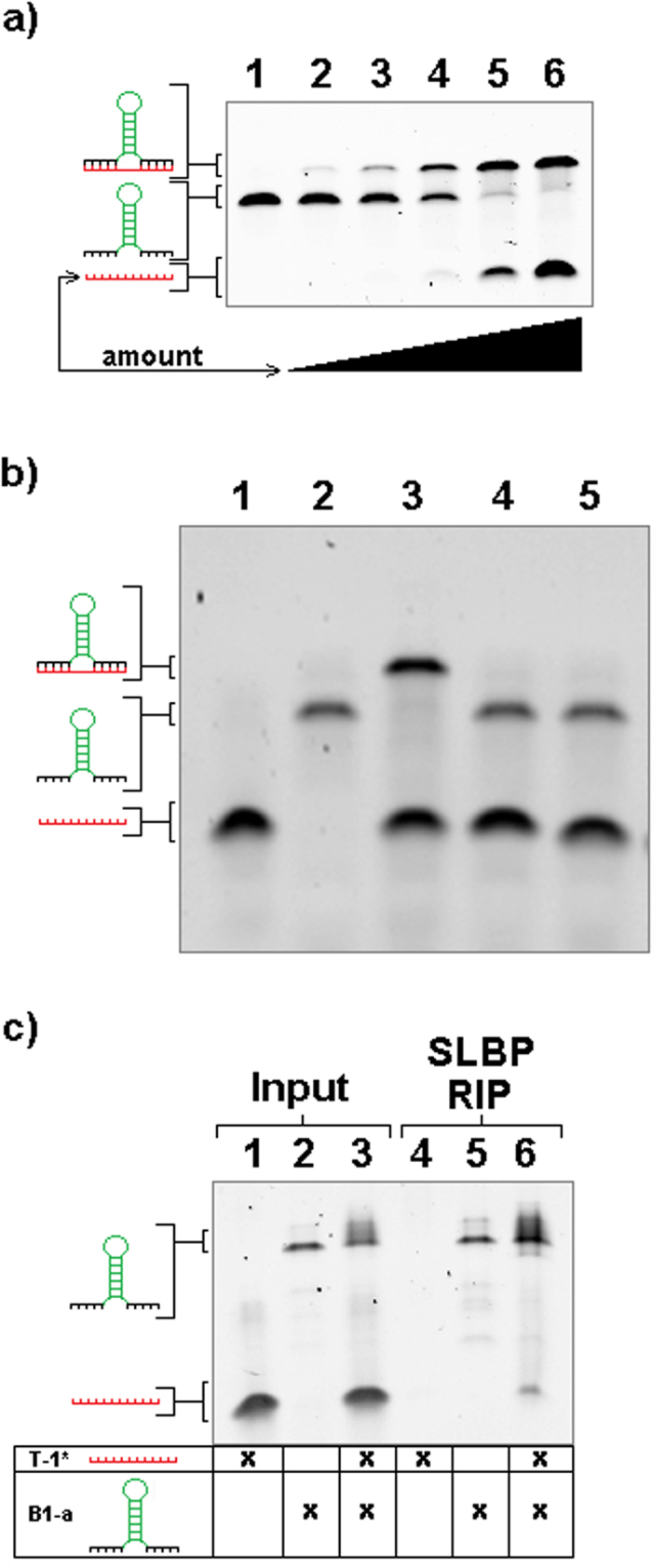
*In-vitro* sxRNA formation and SLBP Binding. (**a**) Trigger Titration and Specificity. Increasing amounts of T1* (serial dilutions) were added to a constant amount of B-1a. All samples were pre-incubated together at 85 °C and allowed to slow cool to room temperature. Samples were then heated to 95 °C in denaturing loading buffer and loaded onto a “partially denaturing” TBE-Urea gel. Lanes (1) B-1a + 0x T1*, (2) B-1a + 1x T1*, (3) B-1a + 2x T1*, (4) B-1a + 4x T1*, (5) B-1a + 8x T1*, (6) B-1a + 16x T1*. (**b**) sxRNA trigger specificity. sxRNA B-1 was combined with three different triggers (T-1*, T-2* and T-3*), pre-incubated together at 85 °C and then allowed to slow cool to room temperature. Samples were then heated to 95 °C in denaturing loading buffer and loaded on a denaturing TBE-Urea gel. Lanes (1) T-1*, (2) B-1a, (3) B-1a + T-1*, (4) B-1a + T-2*, (5) B-1a + T-3* (**c**) SLBP-RIP of sxRNA bait and trigger. sxRNA bait and trigger were incubated with recombinant stem-loop binding protein (SLBP) and then immunoprecipitated using a modified RIP protocol. Post ethanol precipitation the samples are rehydrated, mixed with denaturing buffer and heated to 95 °C and run on a denaturing TBE-Urea gel. Lane 1 input T-1*, (2) input B-1a, (3) input B-1a + T-1*, (4) RIP T-1*, (5) RIP B-1a, (3) RIP B-1a + T-1*.

While informatic predictions were necessary for the initial design of sxRNAs, the actual dynamics of interactions for *trans*- polynucleotides of similar length is difficult to predict with any degree of certainty, and alternate conformations are always possible. To confirm that our engineered sxRNAs would indeed bind in *trans*, the corresponding sxRNA bait and trigger RNAs were incubated together and then heated and slow cooled (heated samples exposed to ambient room temperature [~21C] for 3 minutes) before being run on TBE-Urea denaturing gels. *Trans*-binding of sxRNA bait and trigger was readily observed (representative example, Fig. 2a) and binding often remained intact, even under denaturing conditions. This type of strong 3WJ interaction has previously been noted by others and can remain in as much as 8 M Urea^18^,^21^,^22^. We detected sxRNA bait-trigger binding irrespective of the incubation conditions used, which included various heat-cool strategies and the use of various buffers and cell lysates (data not shown). Further, we found that sxRNA formation was titratable (Fig. 2a) based on the amount of trigger microRNA and specific for the intended microRNA sequence that the bait was designed to interact with (Fig. 2b).

**Figure 3:**
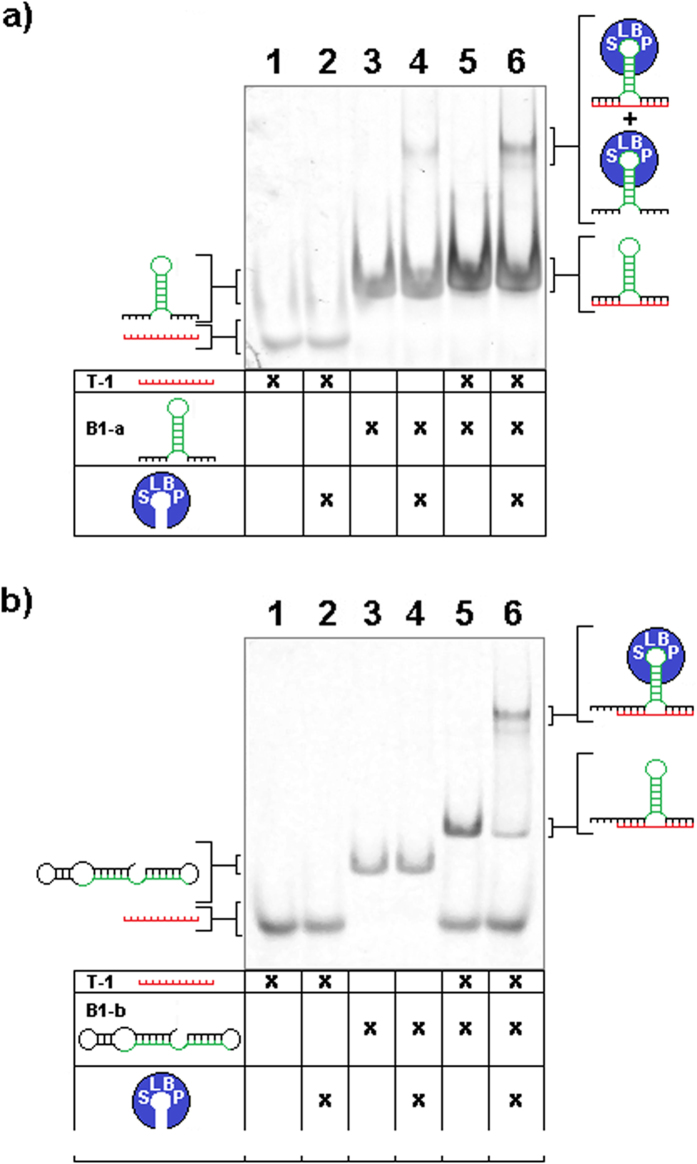
SLBP-EMSA of sxRNA bait and trigger. sxRNA B-1a or B-1b bait 1 μg (~80 and 60 pmol, respectively), trigger (500 ng, ~60 pmol) and combination were incubated with and without 1.25 μg (~37 pmol) recombinant stem-loop binding protein (SLBP), mixed with native loading dye and run on a native 8% polyacrylamide TB gel. (**a**) Lane (1) T-1, (2) T-1 + SLBP, (3) B-1a, (4) B-1a + SLBP, (5) B-1a + T-1, (6) B-1a + T-1 + SLBP (**b**) Lane (1) T-1, (2) T-1 + SLBP, (3) B-1b, (4) B-1b + SLBP, (5) B-1b + T-1, (6) B-1b + T-1 + SLBP.

We next tested if the HSL-motif present in the bait sequence would continue to properly fold in the *trans-*context of the *trans-*3WJ and if SLBP binding would remain possible. We performed a RBP immunoprecipitation (RIP)^24^,^25^,^26^,^27^ modified for use with recombinant SLBP, which was added to the *in-vitro* binding assay described earlier. SLBP binding to the HSL-sxRNA was evident, and co-precipitation of complexed trigger microRNA was observed (Fig. 2c). Further, we tested the ability for recombinant SLBP to bind to the HSL-sxRNA complex and shift its migration in an electrophoretic mobility shift assay (EMSA). We used both a non-switched HSL-bait sequence (Fig. 3a) as well as one that was predicted to only form a proper HSL in the presence of the *trans-*3WJ associated trigger microRNA. Notably, SLBP binding for the switched construct was only observed when the sxRNA complex formed (Fig. 3b). The detailed biochemical analysis of SLBP binding to an HSL formed from a *trans-*3WJ will be the focus of another study.

### Developing an sxRNA-based reporter

**Figure 4:**
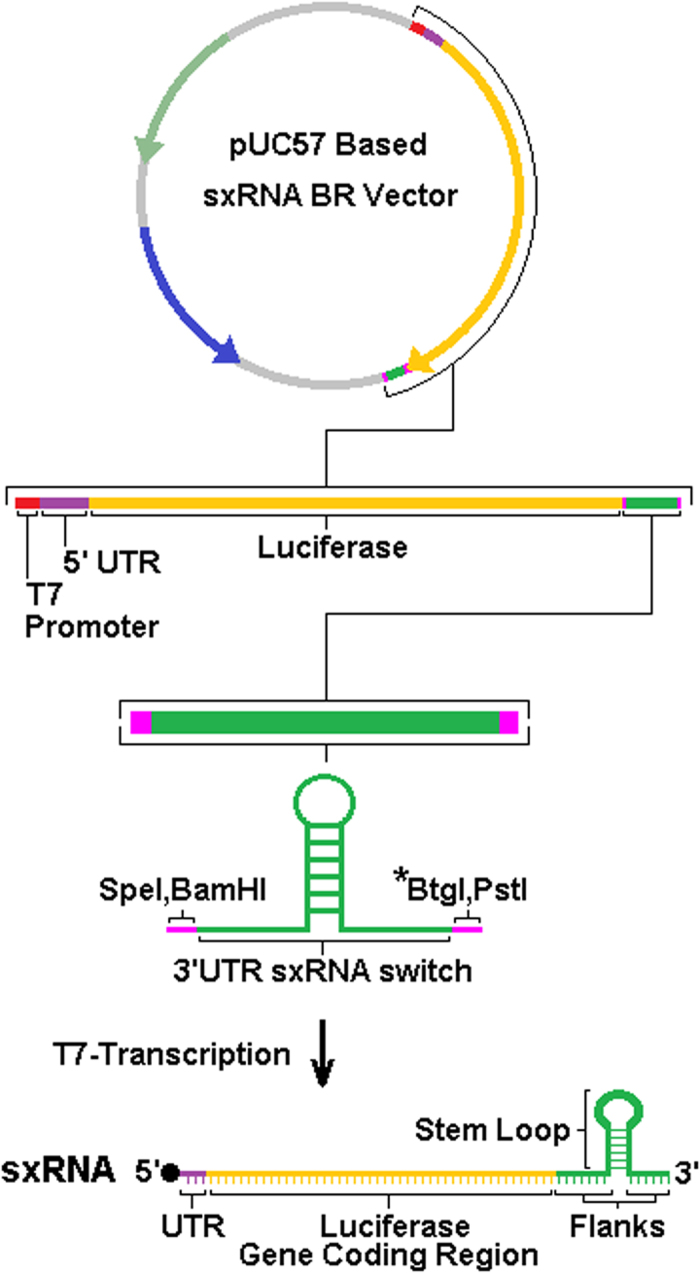
sxRNA Bait-Reporter gene construct. Bait-reporter DNA templates for T7-transcription were created by subcloning designed sxRNA bait and bait-reporter sequences into a pUC57 based plasmid using demarcated restriction sites (GenScript). The region for the insertion corresponds to the 3′UTR of the transcribed, artificial mRNA. Figure 4 depicts vector bait reporter vector 2 (BRV-2), used for all bait reporters other than BR-1a and BR-1b, that both use bait reporter vector 1. BRV-1 has 64 bases 3′ of the luciferase stop codon and 5′ of the switch insert, whereas BRV-2 has only 12 bases in this position and their composition differs from BRV-1 (see Supplemental Data). Restriction site denoted with asterisk is used to linearize plasmid to allow for polymerase “runoff” based termination of transcription. The prototypic architecture of a final, capped, T7-transcribed sxRNA (mRNA) is represented at the bottom.

To determine the potential for the sxRNA technology to be used to regulate the translational activity of gene expression, we designed various plasmids containing HSL-bait sequences in the 3′- UTR of a luciferase gene and downstream of a T7 promoter so the sxRNA could be transcribed into a functional mRNA (Fig. 4). Plasmids were linearized and transcribed using an *in-vitro* T7-capping/transcription system to produce a regulatable luciferase mRNA containing an HSL-bait, sxRNA-sequence at the terminal 3′end (an sxRNA-Bait Reporter). Transcripts were nucleofected into mammalian cell lines in combination with its paired, triggering microRNA and then cells were assessed for luminescence activity.

**Figure 5:**
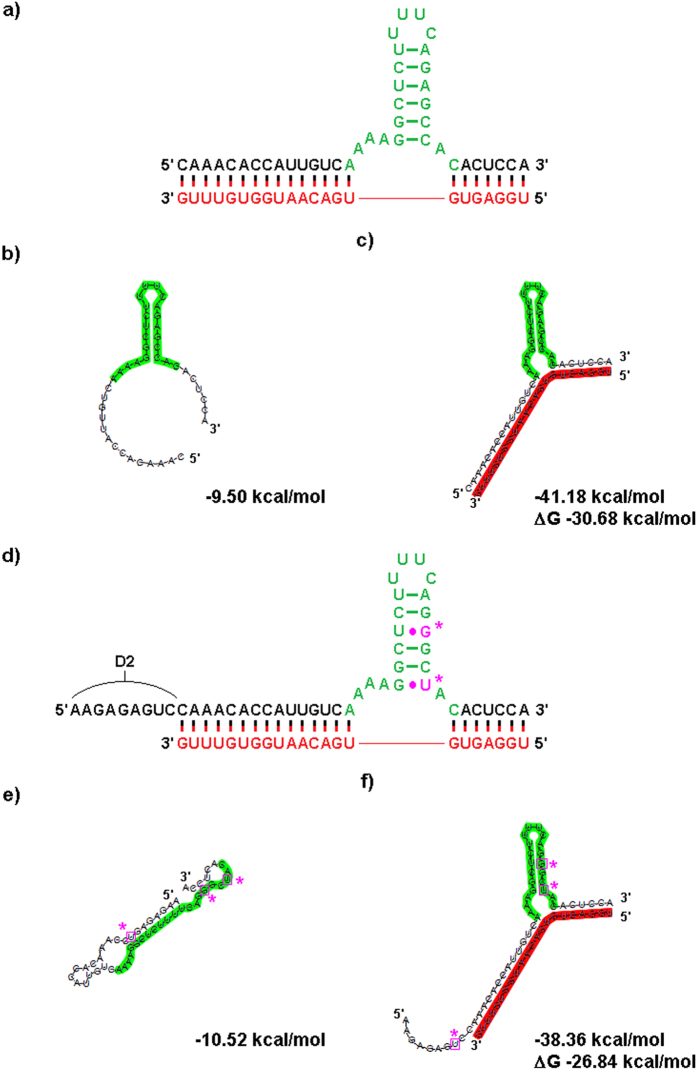
RNA Switch Design and Fold Predictions for B-1a and B-1e sxRNA. (**a**) Design of B-1a, a “non-switched” sxRNA sequence fabricated to interact with trigger T-1 (shown in red). The incorporated histone stem loop sequence is shown highlighted in green. (**b**) RNAfold prediction for the sequence without T-1, note presence of structured HSL motif. (**c**) RNAcofold prediction for sequence with T-1. (**d**) Design of B-1e, a “switched” sxRNA sequence also targeting T-1. The designed 3WJ is identical in position and unpaired region bases as B-1a. However, non-canonical pairings (in purple with asterisks) were engineered into the stem to weaken it and an optional “destabilizing” sequence (“D2”) was appended 5′ of the trigger-pairing region. (**e**) RNAfold prediction for B-1e without T-1, note lack of structured HSL motif. (**f**) RNA-cofold prediction for B-1e with T-1. Note that the structured HSL motif has been predicted to form.

Multiple HSL bait sequences were designed to target the same microRNA trigger sequence, each of which was expected to recruit cellular SLBP when bound to microRNA. We started with a bait sequence that incorporated a stable HSL (predicted to form as part of MFE structure and not considered to be a “switched” construct) with flanks that could base pair to a targeted miRNA and form a 3wj (Fig. 5a–c). Additional sequences were designed that differed with respect to the number of GU base-pairs placed in the HSL motif and the addition of slightly varying “destabilizing” sequences (Fig. 5d and Supplementary Figs S1–S6). These additional sequences were added to promote the formation of structural conformations that would prevent a functional HSL from forming when no microRNA trigger was present and thus produce a “switched” sxRNA with respect to translational activity (Fig. 5e,f and Supplementary Figs S1–S6). Location of the 3WJ relative to the microRNA sequence was kept constant, as were the unpaired bases in each junction region. RNAcofold^23^ was used to design a predicted reduction in the free energy for the bait and trigger interaction versus their independent folds. Additionally, minimum free energy (MFE) structural predictions needed to contain a *trans-*3WJ to be selected for further testing. Specific sequence and nomenclature information is detailed in Table 1.

**Figure 6:**
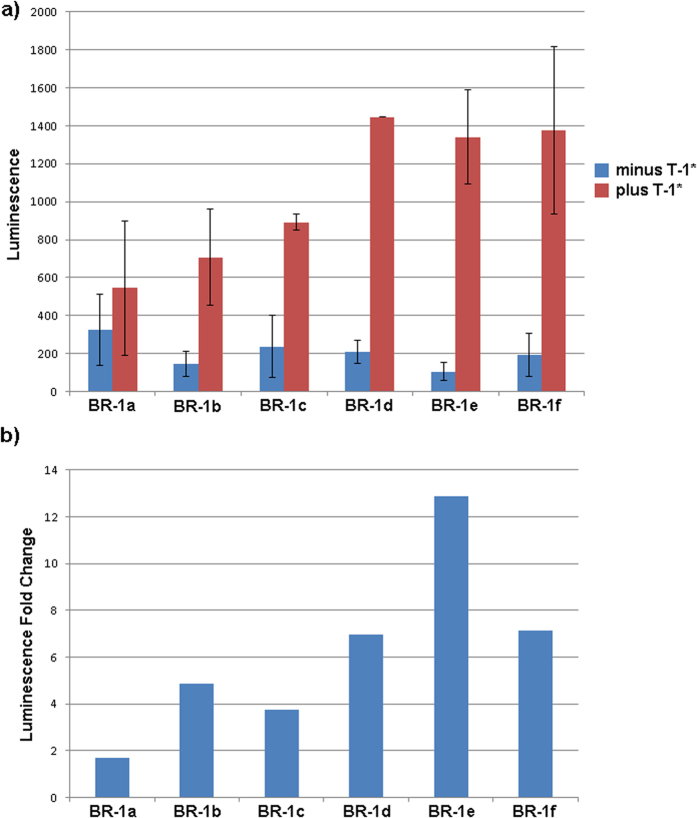
sxRNA trigger induced reporter expression. (**a**) Constant 10 μg of capped BR-1a through BR-1f were nucleofected into 1 million K562 cells with or without accompanying 1 μg of T-1*. Minor differences (see Table 1 and Supplementary Figs 1–6) show varying changes in baseline and “triggered” luminescence. Results are the average of duplicate treatments and error bars show the range of the duplicates. (**b**) The same data as represented in panel A but as fold change in luminescence for triggered switches.

**Figure 7:**
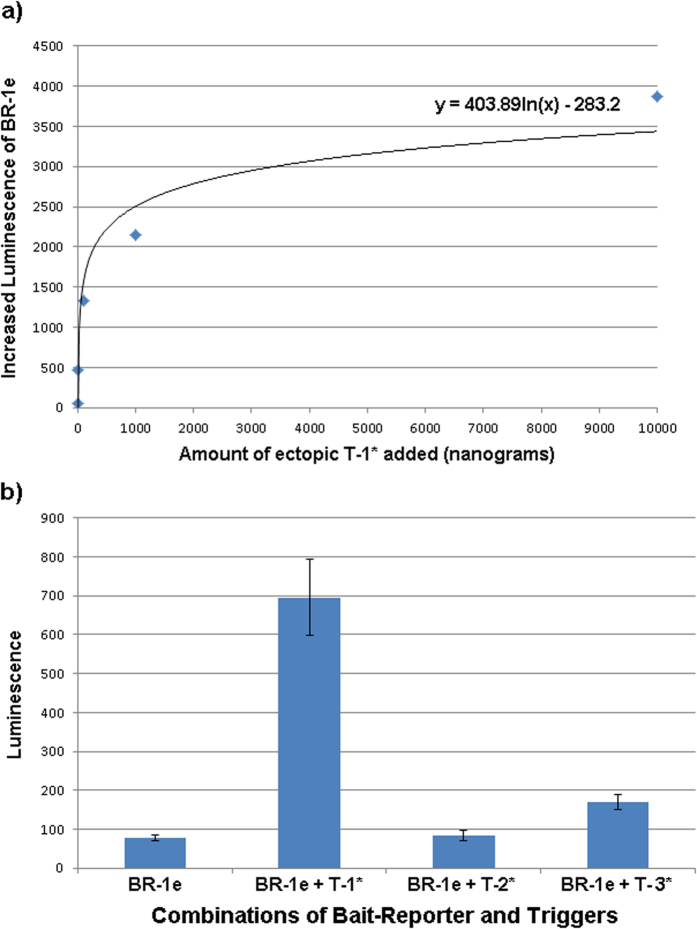
sxRNA fidelity and specificity. (**a**) A constant amount (10 μg, ~18 pmol) of capped BR-1e sxRNA was nucleofected into 1 million K562 cells with 0, 10^0^, 10^1^, 10^2^, 10^3^ and 10^4^ nanograms (~0, 0.136, 1.36, 13.6, 136 and 1360 pmol, respectively) of ectopic T-1*. Plotted values reflecting increases in luminescence from zero T-1* baseline and shows a logarithmic increase of expression. (**b**) A constant amount (10 μg, ~18 pmol) of capped BR-1e sxRNA was nucleofected alone or with 1 μg (~18 pmol) of targeted T-1*, or off targets T-2* and T-3*. Results represent the average of duplicate treatments and error bars show the range of the duplicates.

It should be understood that, while the MFE folds for bait alone and bait complexed with trigger are shown (and used in sxRNA design), these specific conformations are at extremes of a spectrum of potential sub-optimal structures that any given RNA might adopt as it accepts and releases energy from the surrounding system. Single RNA molecules have been shown to reach their most energetically stable conformation by traversing a “rugged energy landscape”, often passing through somewhat stable alternative folds first^28^. The ability of a trigger to change conformation of a bait must involve this plasticity and may require that the bait sample a structure close to its “on” state at some minimal probability to allow for the interaction. Specifically, sxRNA bait-reporters BR-1a through BR-1f were tested by introducing each with or without the targeted trigger microRNA (T-1*) into K562 Human chronic myelogenous leukemia cells. As depicted in Fig. 6, sxRNA bait-reporters showed varying levels of luciferase activity ranging from ~2–12 fold in the presence of the trigger microRNA. Of note, the bait-reporter design showing the greatest range of signal to noise (BR-1e) also demonstrated a strong dose-response with logarithmically elevated luciferase activity in the presence of increasing amounts of trigger microRNA T-1* (Fig. 7a). Further, the targeted T-1* microRNA demonstrated excellent specificity and showed little activity to non-targeted trigger microRNA (Fig. 7b).

It is worth noting that even minimal (1 or 2 base) changes in the bait sequences have substantive effects with regard to expression profiles. For instance, the transition from BR-1c to BR-1d replaces a G-U pairing in the trigger binding region with a G-C and introduces a U to form a predicted G-U pairing in the destabilized “off” structure. The predicted MFE of the “off” state structure changes by only ~1.3 kcal/mol but the ΔG from “off to on” changes by ~3 kcal/mol in favor of the transition. Figure 6a does not show a significant corresponding change in the off state, but does show a marked increase in the “on” signal. As mentioned previously, we avoided altering the location of the 3WJ itself as well as modifying any unpaired junction region bases. Such changes have the potential to introduce tertiary interactions with neighboring helices that are beyond our current ability to predict and that could overcomplicate interpretation of results from a small sample set.

### Development of sxRNA-reporter dependent on an endogenous microRNA for translational activation

**Figure 8:**
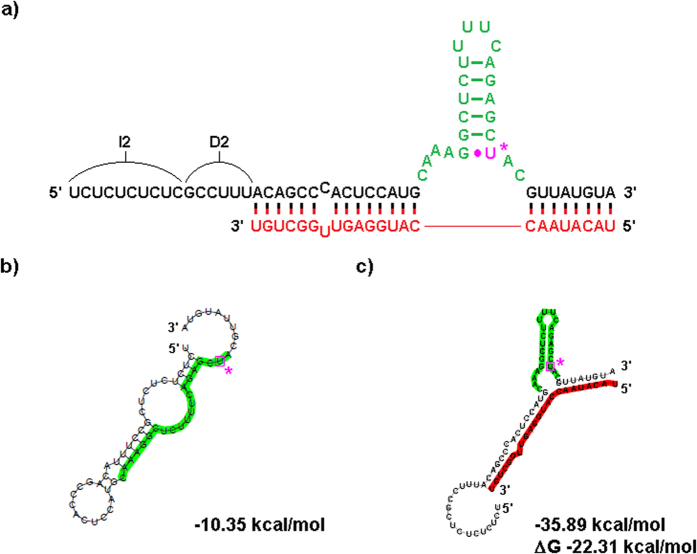
B-5a sxRNA bait-reporter switch design and fold predictions. (**a**) Design of B-5a. A “switched” sxRNA sequence was designed to interact with trigger T-5 (shown in red). The incorporated HSL sequence is shown highlighted in green. This sxRNA includes a sequence intended to act as destabilizer (D2) and a sequence intended to isolate the sxRNA (I2) from preceding 5′ sequence (when in bait-reporter context). Prediction shows both these sequences combining to act as a single destabilizer in the “bait only” context (**b**) RNAfold prediction for the sequence without T-5, note absence of structured HSL motif. (**c**) RNAcofold prediction for sequence with T-5, showing reformed HSL structural motif.

After verifying that sxRNA bait-reporter and corresponding trigger microRNA could bind and produce a functional reporter mRNA that was translationally active when *ectopically* introduced into cells, we assessed the ability of an sxRNA bait-reporter mRNA to be regulated when encountering an *endogenously* produced microRNA. To do this, we designed an sxRNA bait (BR-5a) to target a highly expressed viral microRNA called BART10-3p produced uniquely by the Epstein-Barr virus (designated as T-5 here). This microRNA is produced in abundance by EBV during latency, particularly in the EBV^+^ Burkitt lymphoma cell line, HH514-16. Conversely, the BART10-3p microRNA (T-5) is absent in the lymphoblastoid cell line (LCL), which was generated using the B95.8 strain of EBV^29^ that has this genomic region deleted. Thus, the HH514-16 (T-5+) and the LCL (T-5−) EBV containing cell lines represent an ideal system to evaluate the potential for endogenous microRNAs to specifically trigger the activation of the sxRNA bait-reporter. Using an informatic modeling approach, we designed an sxRNA-bait reporter to be dependent on a T-5 *trans-*3WJ interaction for proper HSL motif formation (Fig. 8).

**Figure 9:**
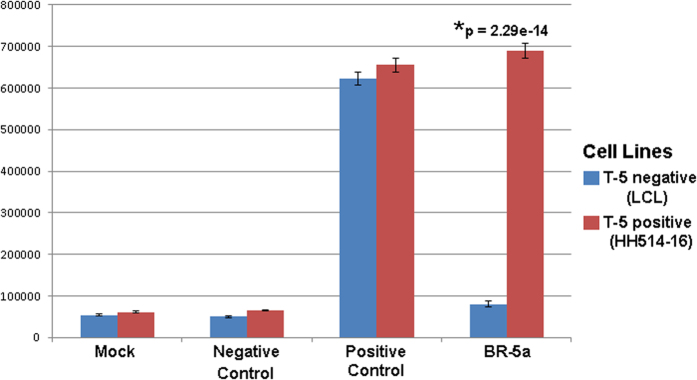
Endogenous microRNA triggers sxRNA activity. Cell lines expressing or not expressing endogenous (EBV) microRNA trigger T-5 were tested for activity with BR-5a sxRNA. Control bait sequences were also used that were designed to have the HSL structure always formed (positive control) or never formed (negative control). Experiments were performed in triplicate and values were not normalized. A two-way (cell type, treatment) ANOVA was applied showing a significant effect for treatment, *F*(3, 16) = 1460, p < 0.001. Post hoc analysis was performed using Tukey’s HSD. Controls treatments did not differ significantly across cell types. BR-5a showed a significant (p = 2.29e-14) increase in expression within cell type expressing T-5. Fold change for BR-5a across cell types ranged from 8.6 (raw mean comparison) to 23.8 (after mock signal subtraction).

Both cell lines expressed similarly high levels of luciferase from a positive control mRNA (Table 1) containing a *cis*-3WJ based sxRNA-HSL, which was designed to be translationally active independent of a trigger microRNA. Conversely, both lines expressed limited levels from a negative control sxRNA (Table 1) that targets the embryonic stem cell specific miR-302. However, the difference in the expression of T-5 dependent luciferase was dramatic between the two cell lines with background levels being produced in the T-5− cells but significantly elevated luciferase expression observed in the T-5+ cells. Thus the ectopic sxRNA translational activity appeared to be regulated by the presence of the endogenously targeted microRNA (Fig. 9).

### Assessing the role of the HSL-motif for functional activity of the sxRNA

**Figure 10:**
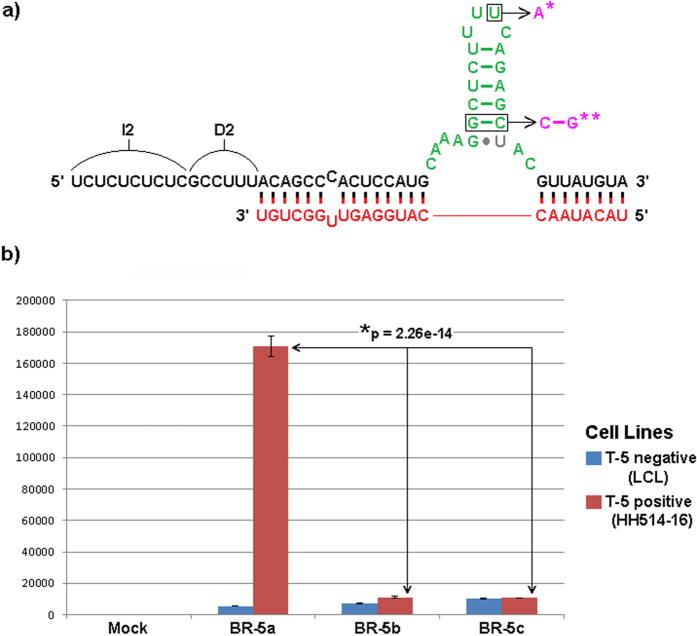
Importance of HSL site on sxRNA activity. (**a**) BR-5a sxRNA shown with point mutations to the HSL known to reduce SLBP binding affinity^43^. BR-5b incorporates the single asterisk (*) noted mutation. BR-5c incorporates both the single asterisk (*) and double asterisks (**) notated mutations. Mutations showed no impact on RNAfold MFE structure predictions for the sxRNA when complexed with trigger and had ΔG changes of less than 7% compared to original BR-5a. (**b**) Experiments were performed and analyzed as described in Fig. 9.

The sxRNA technology presented here depends on the regulatable formation of a functional HSL-motif based on a *trans-*3WJ with the premise that the *trans-*3WJ-HSL, once formed will recruit endogenous SLBP to the 3′UTR of the sxRNA message and facilitate its translation much like it would do with one of the HSL-containing histone mRNAs that are its natural substrate. To assess the significance of the HSL motif, we designed several sxRNAs for the EBV system with mutations in the HSL sequence that should prevent proper SLBP recognition (Fig. 10a). When introduced into the respective T-5− and T-5+ cell lines, only the sxRNA construct with a functional HSL showed translational activity and thus luciferase production, while sxRNAs with mutated HSL sequences showed activity near background levels (Fig. 10b). This clearly demonstrates the sxRNA dependence on the formation of a functional *trans-*3WJ-HSL and the assumed role of SLBP binding effecting translational regulation. Investigations into further details of the nature of this binding and the specifics of SLBP-sxRNA/HSL regulation are ongoing.

## Discussion

In our earlier work we described naturally occurring interactions that resulted from *trans-*3WJ forming between various cellular microRNAs and histone mRNA, specifically at the location of the HSL motif^7^,^8^. We named these complexes sxRNA and proposed that such *trans*-RNA interactions can influence RNA structure and represent a novel mechanism of post-transcriptional gene regulation. In this model, functional sequences or motifs in mRNA could be masked, revealed, or just reinforced in a dynamic and transient fashion. Thus, ncRNA and RBPs would compete with each other to shape the structural, and therefore regulatory, landscape of mRNA.

In the present study, we demonstrated the ability to rationally design an sxRNA-based regulatory switch, in which an mRNA and microRNA (or other ncRNA such as lncRNA or piRNA, etc.), bind to each other in a predictable structural and functional manner. Specifically, we were able to design 3WJs that form and function in *trans* (an sxRNA) and in a highly predictable manner. By incorporating the sxRNA concept into the HSL regulatory motif, we demonstrated that the binding and therefore activity of an RBP (SLBP in this case) could be modulated in a microRNA dependent manner. In this case, the translational level of a tethered upstream reporter gene was regulated. We have also shown that our sxRNA approach can be used to modulate gene expression using an ectopic or endogenous microRNA as the trigger to regulate the availability of a switched RBP motif such as the HSL. Because the HSL is so well characterized and plays such an important role in histone mRNA metabolism and translation, it served as an ideal RBP motif for development of the sxRNA technology. However, its cell cycle regulation could limit its utility and other RBP-regulatory motifs are actively being investigated. Alternatively, the design of sxRNAs based on synthetic aptamers for RBPs may prove more robust. This approach is also more flexible, as it removes the constraint of solely targeting RBPs via their natural motifs and associated recognition domains. Additionally, this strategy could allow targeting of anything a structural RNA aptamer can be developed for (e.g., proteins other than RBPs, small molecules, etc.).

Current informatic tools used for predicting RNA structure, unfortunately often lack the ability to accurately predict either non-canonical base pairings beyond the GU wobble, or resulting tertiary structure. Additionally, our ability to design a switched sxRNA construct currently depends primarily on consensus definitions of known functional structures. Alternatively, optimization of sxRNA switches could be generated using a combinatorial approach such as that provided by SELEX^30^ and this may prove to be a productive complementary design strategy. Although some protein-binding motifs, like the HSL, may be well characterized, including knowledge about the effect of point mutations on SLBP binding, such point mutations in the absence of an sxRNA like reinforcement may actually reduce binding based on structural change rather than lack of protein affinity for the changed base. Counter intuitively, such base substitutions, previously identified as detrimental, might actually improve protein binding when structural context is retained via an sxRNA type interaction. A possible example of this may be apparent in Fig. 6, where bait-reporters incorporating GU pairs in the HSL stem actually show increased overall translation as compared to wild type stems. Although not formally demonstrated, our observed increase in sxRNA-reporter expression is suggestive of enhanced SLBP binding resulting in elevated translation. This could result from various mechanisms including improved interactions, ranging from effects on direct contact of the protein with altered bases to beneficial changes in stem-loop orientation or accessibility of other essential motif contacts, such as those caused by changes in helical twist and bulge formation from non-canonical pairings.

The expression patterns of microRNA have been shown to be distinctively expressed in normal tissues^31^ and in many cancers^32^. By using the unique expression of a cellular microRNA, such as miR-122 in liver cells^33^ or miR-1 in heart cells^34^, sxRNA could be used for the highly specific expression of a strategic protein in a selective tissue. In addition to targeting unique cellular microRNAs to facilitate tissue-specific sxRNA gene expression, other distinctive ncRNAs, such as those produced by pathogens could also be targeted for therapeutic purposes. Several viruses including HSV1, HSV2, CMV, KSHV, HHV6, EBV and Ebola express unique microRNAs that could serve as optimal sxRNA triggers in a manner similar to what we have demonstrated for the EBV microRNA, BART10-3p. Further, by replacing the reporter gene with a death gene, sxRNA might be used as an anti-viral or anti-cancer therapeutic when any unique ncRNA is available to act as the trigger.

It is clear that a better understanding of *trans-*RNA interactions, like the 3WJ as well as the manner in which RBPs interact with these structures, would be beneficial for fully exploiting sxRNAs as a technology. While optimization of a single sxRNA construct may be sufficient, in some cases, better optimization of a switched mRNA might be achieved through the incorporation of multiple discrete sxRNA switches. These switches could redundantly target a single RBP-microRNA interaction or some combination of various RBP-microRNAs. In the case of an aberrantly overexpressed microRNA, a single microRNA could be used as the trigger, or it may be more advantageous to target multiple microRNAs that reflect a particular cell type profile.

We are only beginning to appreciate the vast RNA-RNA interactome and its functional relevance^4^,^5^,^6^. Technologies that are based on these *trans-*interactions, such as sxRNA, represent a powerful new approach with broad applications, especially when coupled with recent advances in RNA delivery methods^35^,^36^,^37^.

## Materials and Methods

### Design of synthetic sxRNA *trans*-3WJ

Representative human HSL sequences were selected (Fig. 1a) and incorporated into defined characteristics for known *cis* 3WJs^1^. We then aligned the HSL with trigger microRNA sequences of interest (see Table 1 and Supplementary Table S1) such that the 3WJ would be closed by strong base pairings with the microRNA on both the 5′ and 3′ sides. The HSL motif has conserved, unpaired flanking sequences both 5′ and 3′ of the stem base consisting of adenosine and cytidine bases. These were utilized as the unpaired “junction/joining region” sequences in their respective positions of the 3WJ. Examples of resulting sequences in predicted conformation with target trigger microRNAs are shown (Figs 1a and 7a).

While some stability of the HSL leading to greater SLBP binding might be conferred to this sequence, we believed a true “switch” with greater dynamic range could be developed. To promote a more reliable “OFF-state” in absence of target microRNA, introducing a G-U pairing at its lowest position was used to weaken the HSL stem and decrease the potential for optimal HSL-motif formation in the absence of the trigger microRNA. Additionally, we added a “destabilizing sequence” at the 5′ of the existing sequence (Figs 1d and 4d to promote an alternate conformation that does not include the HSL structure in the absence of the trigger microRNA. We also occasionally added a short “isolation sequence” consisting of UC repeats to the terminal 5′ region to provide a short non-structured region to separate the “switch” from the remainder of whatever RNA sequence (Fig. 1d) which could be used to make the sxRNA bait component more modular and therefore less likely to interact with unintended sequence in an undesirable manner.

### Cell Culture

Human myeloid leukemia K562 cells (ATCC), EBV-positive Burkitt lymphoma cells HH514-16 and EBV-positive lymphoblastoid cell line (LCL) were maintained at 37 °C under 5% CO_2_ in RPMI 1640 (Flow Laboratories) containing 10% fetal bovine serum (FBS, CELBIO), 50 units/mL penicillin, and 50 μg/mL streptomycin.

### T7-synthesis of sxRNA

DNA template molecules (see Supplementary Table S1 for sequence details) were designed and produced commercially (GenScript, USA). sxRNA vector constructs were linearized using BtgI (New England Biolabs Inc.) following the standard protocol. Linearized DNA was purified using the QIAQuick^®^ PCR Purification Kit (Qiagen) and analyzed for size/integrity and quantity via agarose gel electrophoresis and spectrophotometry (NanoDrop) respectively. 1ug of the purified product was then used as a template for T7 transcription using the MEGAScript^®^ T7 Transcription Kit (Invitrogen) according to the recommended protocol using a 2 hour incubation at 37 °C. The resulting mRNA was TURBO^TM^ DNase treated (Invitrogen) for 15 minutes at 37 °C followed by purification with the MEGAclear^TM^ Transcription Clean-up Kit (Ambion^®^). Concentration of purified message was measure by spectrophotometry (NanoDrop). 60 ug aliquots of purified mRNA were then capped using the ScriptCap^TM^ m7 g Capping System (CELLSCRIPT^TM^) following the recommended protocol with a 1 hour incubation at 37 °C followed by another round of purification via the MEGAclear^TM^ Transcription Clean-up Kit (Ambion^®^).

### *In-vitro* sxRNA binding assay

sxRNA baits sequence and microRNA triggers (Table 1) were designed and commercially purchased (TriLink BioTechnologies). Concentration was analyzed using the spectrophotometer (NanoDrop) and aliquots were made at equal molarities. Equal volumes of sxRNA bait and trigger microRNA were added to the tubes and the total volume brought up to 10 uL with a low salt solution (10 mM Tris/100 mM NaCl). Samples were then heated in a hybridization oven at 85 °C for 5 minutes followed by a slow cool back down to room temperature. Loading buffer was added and each sample was run on a Mini-Protean^®^ TBE (10%) Pre-cast Urea Gel (BioRad) for 1 hour at 150 volts. The gel was then stained with SYBR^®^ Gold Nucleic Acid Gel Stain (Invitrogen^TM^) and analyzed using the VersaDoc^TM^ Imaging System (BioRad).

### sxRNA-SLBP RIP analysis

Plasmid templates containing for the sxRNA-Bait sequences (GenScript) were linearized using Hind-III (New England Biolabs Inc.) following the standard protocol. Linearized DNA was purified using the QIAQuick^®^ PCR Purification Kit (Qiagen) and analyzed for size/integrity and quantity via agarose gel electrophoresis and spectrophotometry (NanoDrop) respectively. 1 ug of the purified product was then used as a template for T7 transcription using the MEGAshortscript^TM^ T7 Kit (Invitrogen^TM^) according to the recommended protocol using a 1-hour incubation at 37 °C. The resulting mRNA was TURBO^TM^ DNase treated (Invitrogen) for 15 minutes at 37 °C followed by phenol/chloroform extraction and ethanol precipitation. Trigger-microRNA was designed and purchased commercially (TriLink BioTechnologies) and concentration was analyzed using the spectrophotometer (NanoDrop). 3 ug of microRNA-trigger and 1 ug of the sxRNA bait were used for each RIP sample and the total volume was brought up to 10 uL with low salt solution (10 mM Tris/100 mM NaCl) as necessary.

SLBP-RIPs were performed using previously published methods^24^,^25^,^26^,^38^,^39^. Briefly, tubes containing sxRNA bait and trigger were then incubated at 85 °C for 5 minutes in a hybridization oven followed by a slow cool back down to room temperature. Samples were then incubated at 37 °C for 1 hour after being mixed with 2.5 ug of SLBP protein, BSA (5% concentration) and were brought up to 100 uL with (1x) NT-2 Buffer (Tris, NaCl, MgCl_2_, NP-40, Di H_2_O). During this incubation, the DYNABEADS^®^ Protein G magnetic beads (Novex by Life Technologies) were washed and tumbled with 5 ug of SLBP monoclonal antibody (Abgent) for 1 hour at room temperature. The beads were then washed with NT-2 buffer, re-suspended in NET-2 buffer (NT-2, EDTA, DTT, RNase OUT^TM^) and mixed with the RNA/protein mix. The tubes were then allowed to tumble overnight at 4 °C. Each sample was washed with cold NT-2 buffer and then re-suspended in Proteinase K buffer (NT-2, SDS, Proteinase K) followed by an incubation at 55 °C for 30 minutes. A phenol/choloroform extraction followed by ethanol precipitation (NH_4_OAc, LiCl, Glycogen, Ethanol) was then performed on each tube. Samples were re-suspended in 10 uL low salt solution (10 mM Tris/100 mM NaCl) and mixed with Gel Loading Buffer II (Ambion^®^). Tubes were heated at 95 °C for 5 minutes and spun briefly before loading warm into a Mini-Protean^®^ TBE-Urea denaturing Gel (BioRad) and run for 1 hour at 150 volts. The gel was then stained with SYBR^®^ Gold Nucleic Acid Gel Stain (Invitrogen^TM^) and analyzed using the VersaDoc^TM^ Imaging System (BioRad).

### sxRNA-SLBP EMSA analysis

Individual and mixed samples of 500 ng T-1 RNA (synthezised by Trilink) and 1 ug B-1a or B-1b sxRNA (T7 transcribed from linearized plasmid template as previously described) were heated to 85 °C in 40 uL volume of 1x NT2 buffer, then allowed to slow cool. Volumes were then brought to 50 uL, retaining 1x NT2 concentration, adding 10 units of RNaseOUT^TM^, and brought to 1 mM DTT. 1.25 ug of Recombinant SLBP were added where noted. All samples were incubated at room temperature for 1 hr, mixed with native loading dye, and run on a native 8% polyacrylamide TB gel with 1x TB buffer for 1 hr at 100 volts. Gel was stained with SYBR^®^ Gold and analyzed with the VersaDoc^TM^ System.

### Endogenous sxRNA bait-reporter nucleofection assay

Aliquots (10 ug) of the T7 transcribed mRNA (sxRNA luciferase reporter) were used for nucleofection (Amaxa 4D) either alone or mixed with 1 ug of the microRNA trigger. All samples were heated in a hybridization oven at 85 °C for 5 minutes followed by a slow cool back down to room temperature. During this time, the K562 cells were spun down and old media was removed. The SF nucleofection solution (Lonza) was added to the cell pellet per the standard protocol and then aliquots containing 1 million cells were added to the RNA samples before being place in the nucleofection cuvettes. After nucleofection (program FF120), fresh media was immediately added to the cuvette to dilute the nucleofection buffer. All samples were then plated out in 6 well plates containing pre-warmed media. 4-hours post nucleofection, samples were spun down in tubes, media was exchanged, followed by the addition of the two buffers associated with the luciferase assay (ABI). Samples were loaded into a 96-well plate and allowed to incubate, protected from light for 15 minutes. During this incubation, the plate reader (TECAN) was warmed up to the recommended temperature (25 °C) and the program was loaded. Post incubation the plates were analyzed for luciferase signal.

### Endogenous sxRNA testing in EBV positive cells lines

HH514-16 cells and LCL (lymphoblastoid cell lines) were transfected with sxRNA as previously described^40^. Briefly, sxRNA material was introduced into 1 × 10^6^ cells by nucleofection using an Amaxa Nucleofector II^41^, cells were harvested 4 to 6 hours later, and assayed in triplicate for luciferase expression using a FLUOstar Optima plate reader (BMG Labtech).

For HSL mutation analysis studies, HH514-16 cells and LCL were subcultured at 3 × 10^5^ cells/ml and 5 × 10^5^ cells/ml, respectively 24 h prior to transfection and washed twice with phosphate-buffered saline (PBS). sxRNA was introduced into 1 × 10^6^ cells in 100 μl of electroporation solution (Ingenio electroporation solution, catalog number MIR 50117; Mirus Bio). Cells were electroporated using an Amaxa Nucleofector II (A024 program) and then diluted to 3 × 10^5^ cells/ml in complete RPMI 1640. Cells were harvested 6 hours later and assayed in triplicate for luciferase expression using a Lumat LB 9507 (Berthold Technologies).

## Additional Information

**How to cite this article:** Doyle, F. *et al*. Engineering Structurally Interacting RNA (sxRNA). *Sci. Rep.* **7**, 45393; doi: 10.1038/srep45393 (2017).

**Publisher's note:** Springer Nature remains neutral with regard to jurisdictional claims in published maps and institutional affiliations.

## Supplementary Material

Supplementary Figures

Supplementary Table S1

## Figures and Tables

**Table 1:** sxRNA Bait and Trigger Sequences Used.

Name in Manuscript	RNA Sequence (switch region only in bait-reporters)
Triggers	
T-1	UGGAGUGUGACAAUGGUGUUUG
T-1*	UGGAGUGUGACAAUGGUGUUUG
T-2*	UGGAAUGUAAAGAAGUAUGUAU
T-3*	UGAGGUAGGAGGUUGUAUAGUU
T-4*	GUCCAGUUUUCCCAGGAAUCCCU
T-5	UACAUAACCAUGGAGUUGGCUGU
Bait	
B-1a	GGGCAAACACCAUUGUC**AAAAGGCUCUUUUCAGAGCCAC**ACUCCA
B-1a*	CAAACACCAUUGUC**AAAAGGCUCUUUUCAGAGCCAC**ACUCCA
B-1b	GGGAAGAGAGCCCAAACACCAUUGUC**AAAAGGCUCUUUUCAGAGCUAC**ACUCCAGAGCU
Bait Reporters	
Positive Control	UCAUUUAGUGAAAACUAAAUGAA**AAAAGGUCCUUUUAAGGACCAA**AAU
BR-1a	CAAACACCAUUGUC**AAAAGGCUCUUUUCAGAGCCAC**ACUCCA
BR-1b	GAAGAGAGCCCAAACACCAUUGUC**AAAAGGCUCUUUUCAGAGCUAC**ACUCCA
BR-1c	AAGAGAGCCCAAAUACCAUUGUC**AAAAGGCUCUUUUCAGAGCUAC**ACUCCA
BR-1d	AAGAGAGCUCAAACACCAUUGUC**AAAAGGCUCUUUUCAGAGCUAC**ACUCCA
BR-1e	AAGAGAGUCCAAACACCAUUGUC**AAAAGGCUCUUUUCAGGGCUAC**ACUCCA
BR-1f	CAAACACCAUUGUC**AAAAGGCUCUUUUCAGGGCUAC**ACUCCA
BR-5a	UCUCUCUCUCGCCUUUACAGCCCACUCCAUG**CAAAGGCUCUUUUCAGAGCUAC**GUUAUGUA
BR-5b	UCUCUCUCUCGCCUUUACAGCCCACUCCAUG**CAAAGGCUCUUUACAGAGCUAC**GUUAUGUA
BR-5c	UCUCUCUCUCGCCUUUACAGCCCACUCCAUG**CAAAGCCUCUUUACAGAGGUAC**GUUAUGUA
Negative Control	UCACUAAAACAUG**CAAAGGCUCUUUUCAGAGCUAC**GAAGCACUUA
