# Supplementary material for: Engineering Structurally Interacting RNA (sxRNA)
Source: Sci Rep. 2017 Mar 28;7:45393. doi: 10.1038/srep45393 (PMC5368982; doi:10.1038/srep45393)

## Supplementary Figures

# Engineering Structurally Interacting RNA (sxRNA)

Francis Doyle[1], Sameer Lapsia[2+], Salvatore Spadaro[2], Zachary E. Wurz[3], Sumita Bhaduri-McIntosh[4] and Scott A. Tenenbaum[1]*

**Supplementary Figures S1-S6. RNA Switch Design and Fold Predictions for B-1a through B-1f sxRNA.** In all cases: (**A**) Design of sxRNA sequence fabricated to interact with trigger T-1 (shown in red). The incorporated histone stem loop sequence is shown highlighted in green. Bases colored purple and denoted with asterisk, if present, highlight internal changes made from the core BR-1a sequence and to the destabilizer sequence introduced in BR-1b. (**B**) RNAfold prediction for the sequence without T-1. (**C**) RNAcofold prediction for sequence with T-1. ΔG value is the difference of the heterodimer structure's energy and the sum of the monomers' individual fold energies.

**Supplementary Figure S1**

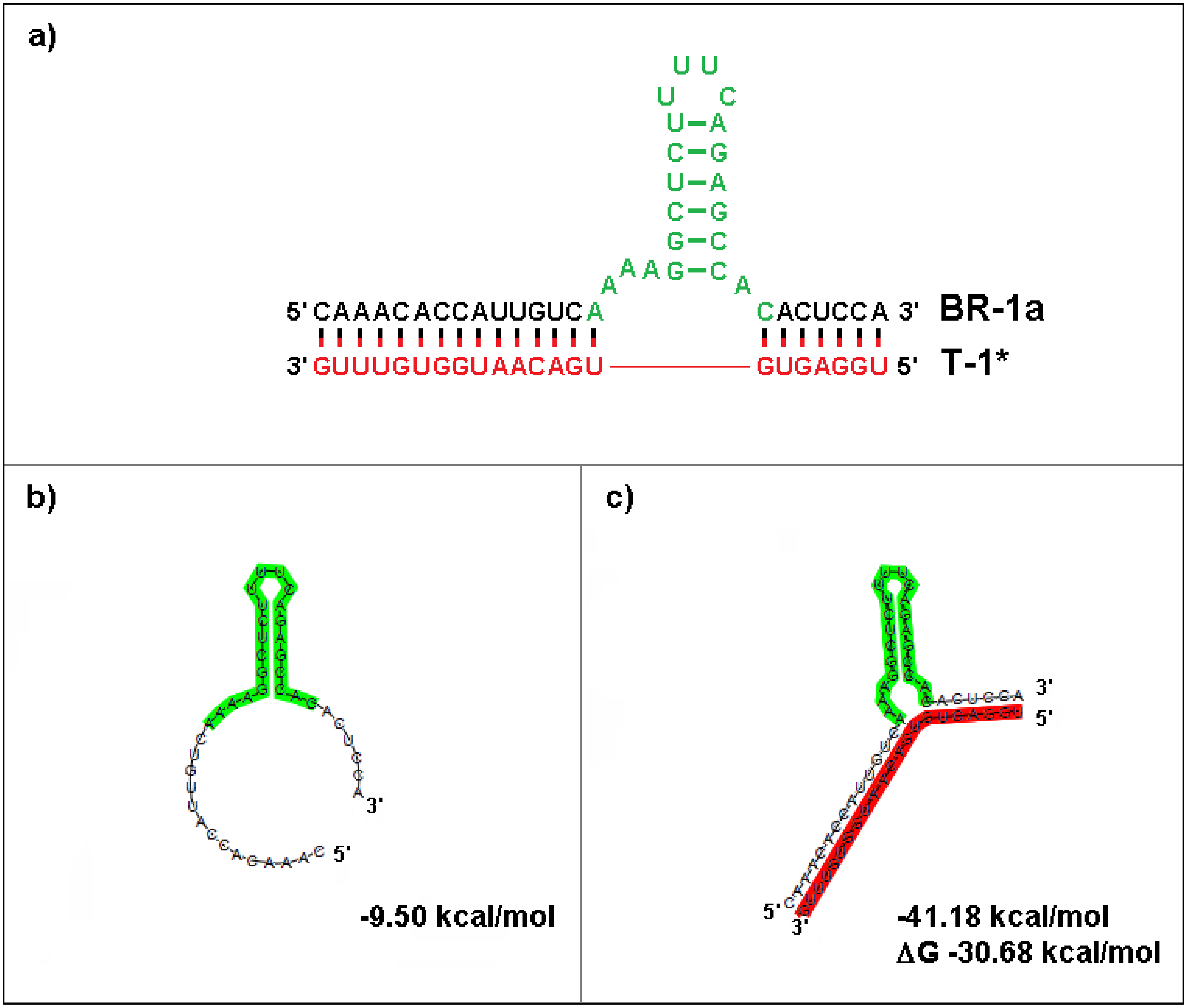

## Supplementary Figure S2

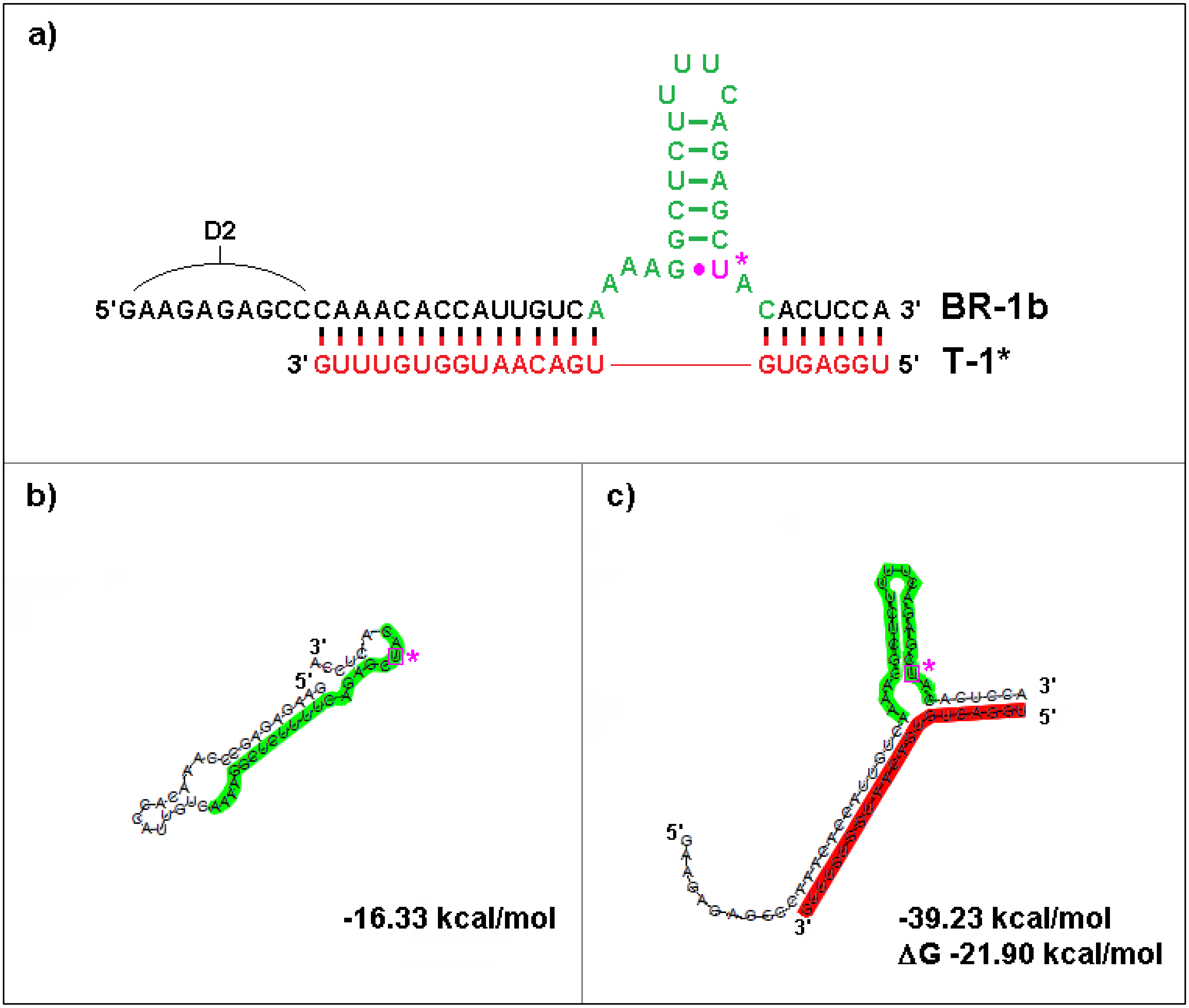

**Supplementary Figure S3**

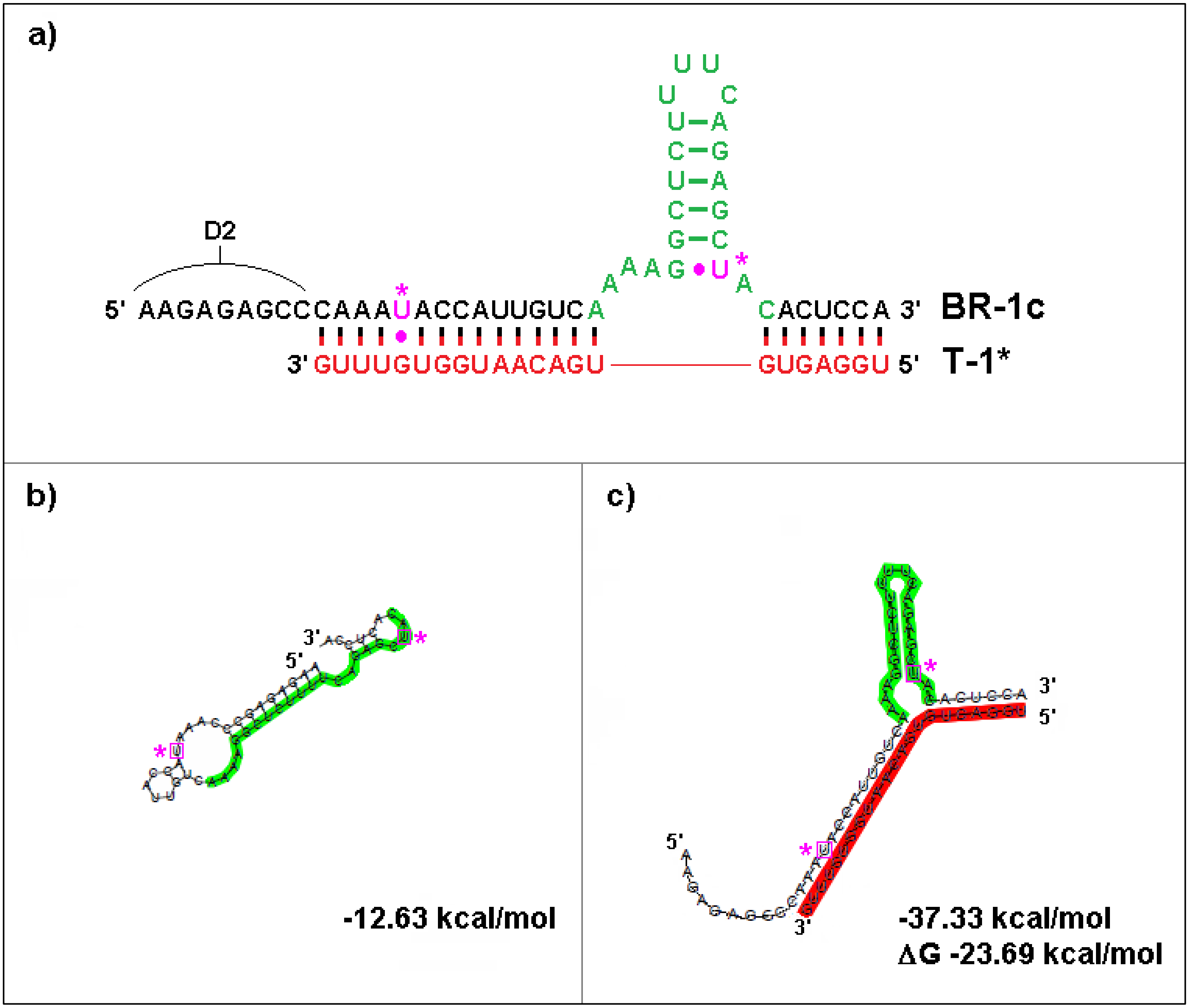

**Supplementary Figure S4**

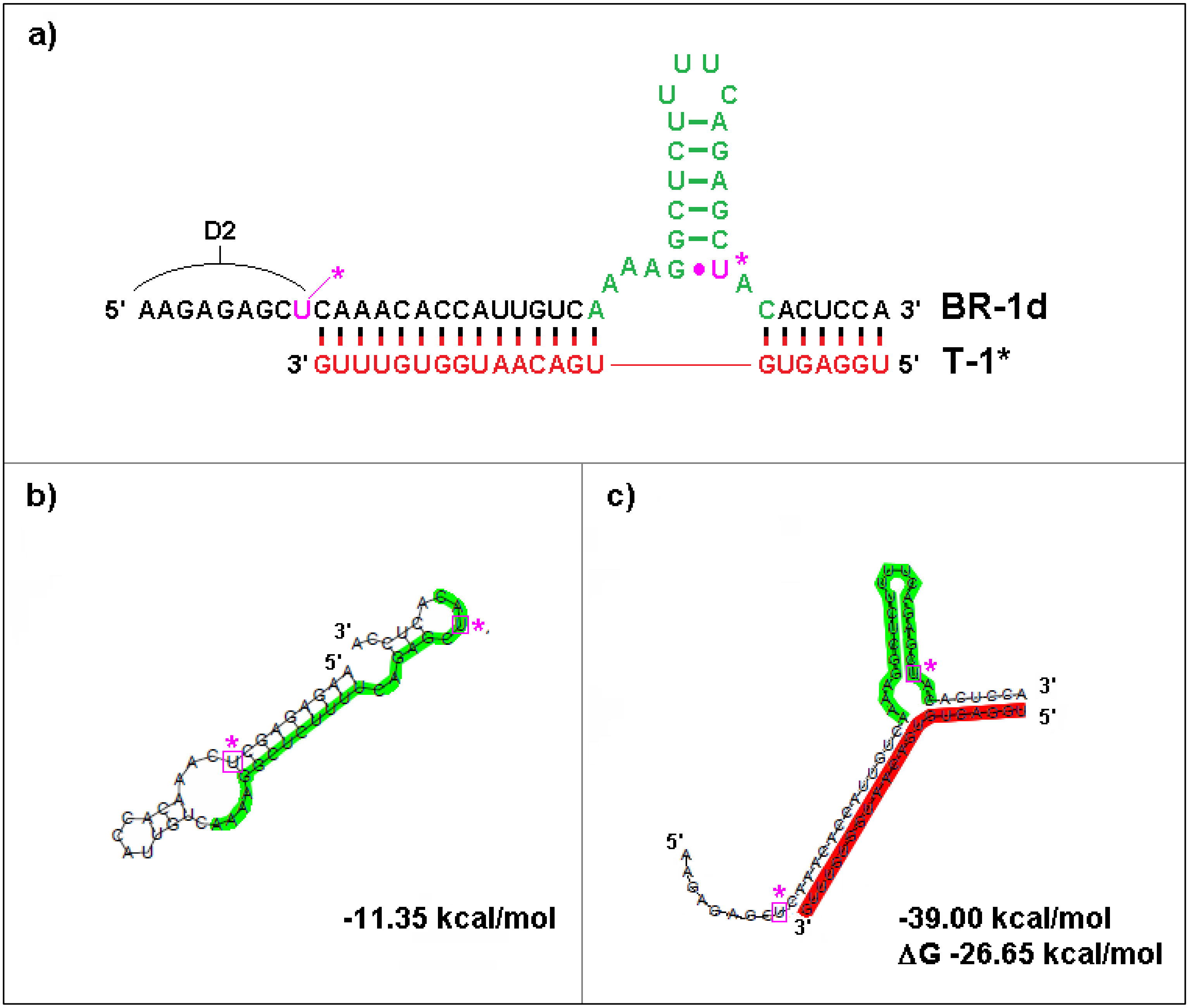

**Supplementary Figure S5**

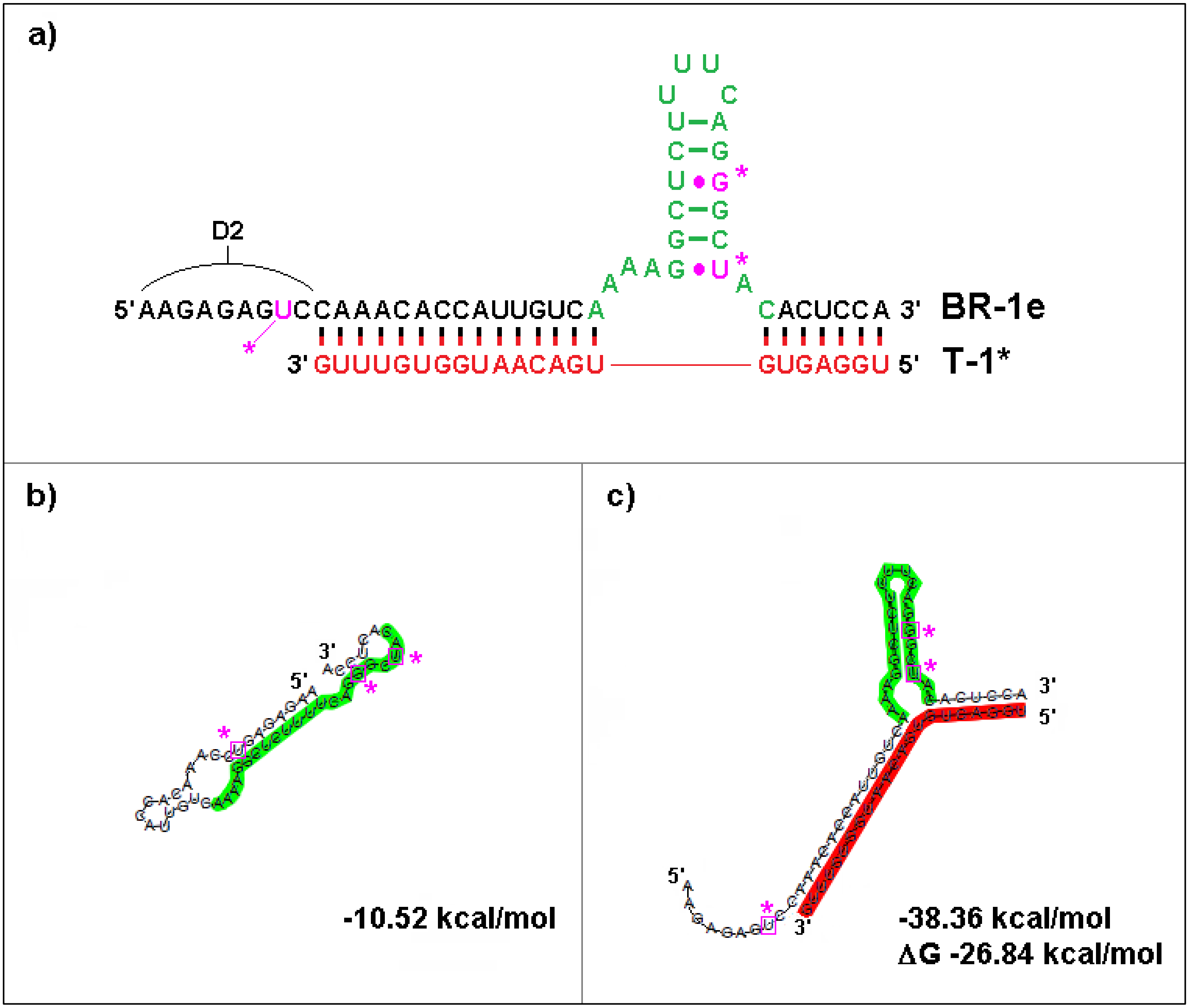

**Supplementary Figure S6**

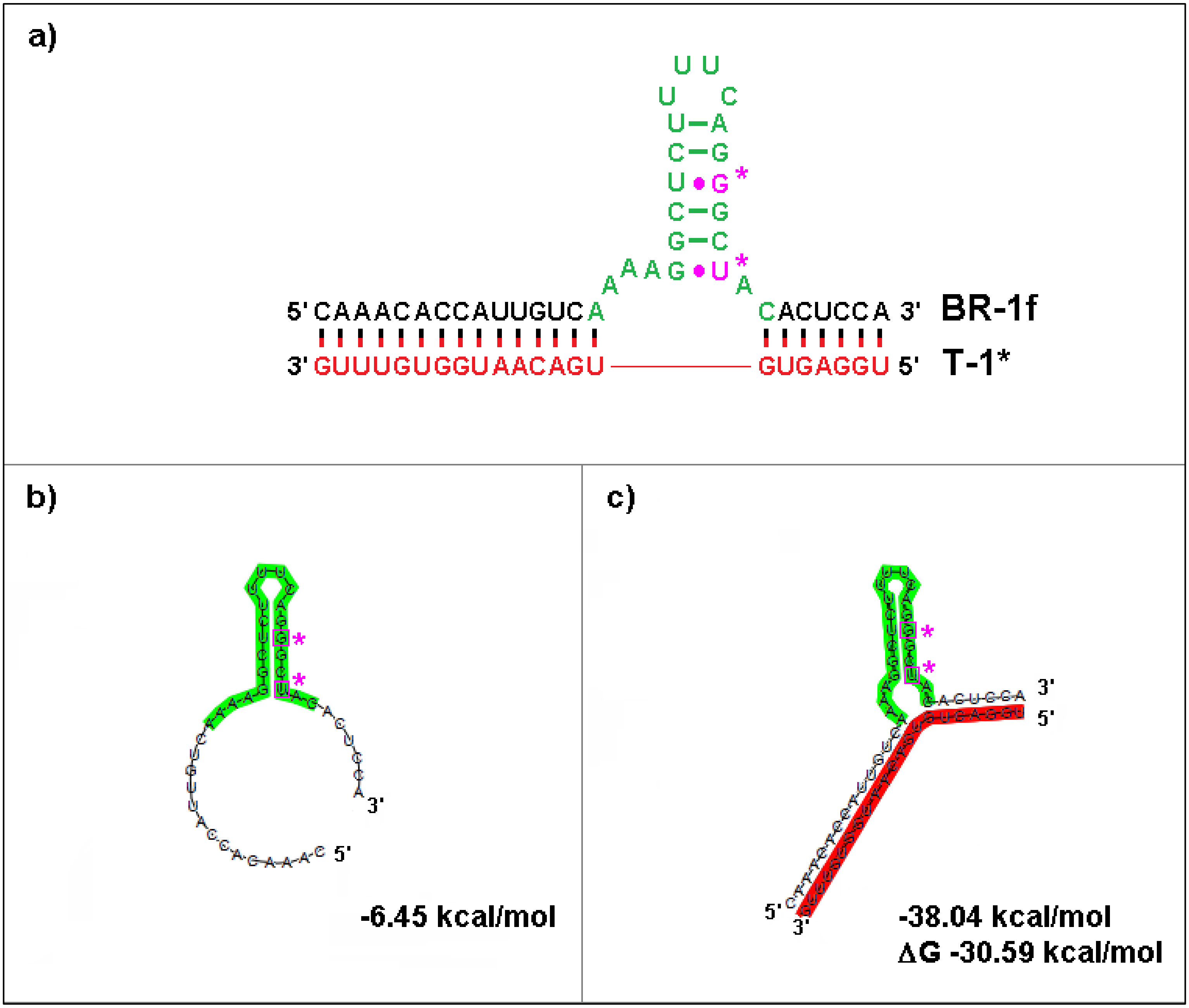

Supplement: Supplementary Figures [file srep45393-s1.pdf]
